# Prognosis and immunotherapy response prediction based on M2 macrophage-related genes in colon cancer

**DOI:** 10.1007/s00432-023-05573-6

**Published:** 2024-01-25

**Authors:** Xiaochen Xu, Xinwen Zhang, Ruilong Kou, Yihao Liu, Siqi Chen, Zuguo Li, Zhiyuan Jian, Zhenran wang

**Affiliations:** Department of Gastrointestinal Surgery, Affiliated Hospital of Guilin Medical University, Guangxi Zhuang Autonomous Region, Guilin, 541001 China

**Keywords:** Colon cancer, Prognosis, M2 macrophage, Immune infiltration, Immunotherapy

## Abstract

**Background:**

M2 macrophage were revealed to play a crucial role in immune evasion and immunotherapies. This study aims to explore the potential significance of M2 macrophage-related genes in colon adenocarcinoma (COAD) by analysizing the transcriptome data in a comprehensive way.

**Methods:**

We collected RNA-sequencing (RNA-seq) data of COAD from The Cancer Genome Atlas (TCGA) and Gene Expression Ominibus (GEO) databases. We calculated the immune infiltration scores of every sample using CIBERSORT algorithm. Through weighted gene co-expression network analysis (WGCNA), we picked out M2 macrophage-related genes. With these genes we screened out prognosis related genes which were utilized to construct a signature to assess the prognosis of patients. To extend the potential application of the signature, we also calculated the correlations with immune infiltration. Finally, we applied techniques such as quantitative reverse transcription polymerase chain reaction (qRT–PCR) and immunoblotting (Western Blotting) to validate the RNF32 gene in cellular in vitro assays.

**Results:**

Seven M2 macrophage-related genes signature was constructed, which was an excellent prognostic predictor in two independent groups. The high-risk group showed lower immune infiltration and poorer response to immunotherapies than those of the low-risk group. The cell vitro experiments showed that the expression level of RNF32 was upregulated in colon cancer cell lines compared with normal cell lines. Moreover, we found that RNF32 may promote the proliferation, migration and invasion of cancer cells in vitro by inhibiting apoptosis.

**Conclusion:**

A novel M2 macrophage-related gene signature affects the prognosis and immune characteristics of colon cancer.

**Supplementary Information:**

The online version contains supplementary material available at 10.1007/s00432-023-05573-6.

## Introduction

As one of the most common malignancies, colon cancer accounts for approximately 10% of all tumor mortality worldwide (Sung et al. 2021). Currently, combination treatments of surgical resection and chemotherapies have contributed enormously to significantly enhanced survival of patients (Dehal et al. 2018). However, the deficiency of clinical treatments and the increasing mortality call for in-depth understanding of the mechanistic underpinning and molecular features of colon cancer. Besides, clinical and histopathological characteristics of tumors could not always predict patients' outcomes accurately due to tumor heterogeneity.

Over the past decade, the notions and scopes of immunotherapies have been solidified and extended. Also, several immunotherapies have been approved for cancer treatment, including immune checkpoints inhibitors (ICIs) (Robert 2020) and chimeric antigen receptor T (CAR-T) cell therapy (Miliotou and Papadopoulou 2018), etc. Though various immunotherapies have been successfully applied, it is critical to note that many patients are subjected to resistance to immunotherapies and only a small proportion of patients benefit from them (Ott et al. 2013). Successful immunologic elimination of cancer cells relies on further investigatation of the interplay between the immune system and tumor cells. Also, immune escape to ICIs might be a notable problem, whose molecular mechanisms have been far from elucidation.

Previous researches have indicated that evasion to checkpoint inhibitors or other immunotherapies might be mediated by infiltrated tumor-associated macrophages (De Henau et al. 2016; Kaneda et al. 2016). Traditionally, macrophage could differentiate into two phenotypes: M1 and M2 in different microenvironment (Wang et al. 2019), which are mainly involved in pro-inflammatory and anti-inflammatory responses respectively (Murray et al. 2014). Besides, it is generally believed that the recruitment and infiltration of M2 macrophage in tumors might contribute to the immune escape of tumor cells, and thereby advance tumor progression and metastasis (Ho and Liu 2016). Moreover, “re-educating” M2 macrophage to repolarize into M1 macrophage has shown potential in immunotherapies (Ho and Liu 2016). Therefore, the genes related to M2 macrophage are promising therapeutic biomarkers and drug targets in the clinical practice.

Moreover, as omics data rapidly accumulate, the translation of these data into clinical application might be a major challenge and also a favorable opportunity for personalized medicine. Herein, we screened out M2 macrophage-related genes and investigated their clinical application at a transcriptome level. We also provided some references for in-depth molecular characterization of macrophage polarization, which has seldom been reported to date.

## Materials and methods

### Data collection

From the official website of TCGA (https://portal.gdc.cancer.gov/repository), we downloaded the RNA-Seq data along with relevant clinical information of 398 COAD tumor and 39 normal samples. The validation dataset was downloaded from the GEO database (http://www.ncbi.nlm.nih.gov/geo/, GSE17536) (Smith et al. 2010) which contains gene expression microarray and clinical information of 177 COAD patients. Finally, we normalized the RNA expression level of the two datasets using “limma” R package.

### Immune cell infiltration and weighted gene co-expression network analysis

We calculated immune infiltration scores with the CIBERSORT algorithm (Newman et al. 2015). Then we compared the differences between normal and tumor samples. We also performed weighted gene co-expression network analysis (WGCNA) to figure out M2 macrophage-related genes. First, we created a co-expression network with the expression data of TCGA dataset and “WGCNA” R package. We selected 150 as the cutHeight to remove the outliers. Second, similar genes were classified into the same module with an optimal softPower (Zhang et al. 2018). Subsequently we calculated the correlations between modules and immune cells infiltration. Genes in these corresponding modules significantly correlated with M2 macrophages were extracted for subsequent analyses.

### Construction and validation of M2 macrophage-related gene prognostic model

We divided patients from the TCGA dataset into training and test cohorts by seven to three randomly. Then, we conducted univariate cox regression to screen out prognostic genes in the training cohort. We conducted the least absolute shrinkage and selection operator (LASSO) regression to construct a prognostic signature. We also plotted Kaplan–Meier (KM) survival curves of the genes in the signature with the “survival” R package. Based on the median riskscore of the training cohort, patients from the three cohorts were assigned to high-risk and low-risk groups separately. Risk score could be calculated by ∑ (expression * βi), β was the coefficient of every gene in the signature. We validated the signature with the KM curves. We also calculated and displayed the 1-, 3-, 5-year receiver operating characteristic (ROC) curves of the signature with “time-ROC” R package. We calculated the 1-year ROC curves of such clinical traits as age, gender, stage and compared them with that of the signature.

In TCGA cohort, univariate and multivariate Cox proportional hazards analysis were carried out to explore the efficacy of the riskscore when incorporated into clinical traits (age, gender, stage). We calculated the correlations of the riskscore with the survival statuses of patients in TCGA cohort. We also compared the expression levels of the genes in the signature between different risk groups. In addition, we displayed clinical traits of different risk groups and calculated the differences. Age, stage, riskscore met the criteria and were utilized to develop a nomogram to predict outcomes with the “rms” R package. We evaluated the validity of the nomogram using the Hosmer–Lemeshow test.

### Exploration of the correlations between immune infiltration and the signature

As the signature was constructed based on M2 macrophage-related genes, we explored the correlations between immune infiltration and the riskscore. We calculated the immune score, stromal score and ESTIMATE score of every sample in TCGA dataset with the “estimate” R package (Galon et al. 2012). Then we displayed the differences between different risk groups. By collecting the checkpoint genes from previous studies, we compared the expression levels of checkpoint genes between high-risk and low-risk groups, and calculated and presented the correlations of the riskscore with checkpoint genes.

### Differences of immunotherapy responses between the groups

To further explore the potential of the signature in immunotherapies, we obtained the Immunophenoscores (IPS) of COAD patients from The Cancer Immunome Database (TCIA, https://tcia.at/home) (Charoentong et al. 2017). Patients with higher IPS are more likely to respond to immune-checkpoint inhibitors. We compared the IPS between different risk groups.

### Expression verification signature genes

Gene expression profiling interactive analysis (GEPIA), a widely used online database, which contains RNA-sequencing expression data of tumor and normal samples from TCGA and genotype tissue expression projects (GTEx). GEPIA provides online interaction and customization analyses for tumor expression profiling as well as expression profiling of normal tissues (Tang et al. 2017). In this study, we used GEPIA to compare the mRNA expression levels of signature genes in COAD and normal tissues.

### Cell culture

Human normal intestinal epithelial cells (NCHM460) were obtained from IMMOCELL in Xiamen, China, and human colon cancer cell lines (Caco2, HCT15, HCT116, HT29, Lovo, SW480, SW620) were obtained from icell in Shanghai, China. All these cells were cultured in media containing 10% fetal bovine serum (FBS) and 1% penicillin/streptomycin (P/S) at 37 °C in a humidified atmosphere of 5% CO2. The culture media used were DMEM, MEM, 1640, McCOY’s 5A, McCOY’s 5A, F12K, L15 and L15, which were purchased from Gibco BRL in the USA.

### Overexpressing RNA and siRNA knockdown

SW480 or Caco2 cells were seeded at a density of 3.0 × 10^5^ cells per well in a six-well plate. Transfection was performed using Lipofectamine 2000 reagent (BL623A, Biosharp) with pre-engineered human overexpression of PCDH-RNF32, RNF32 siRNA, or negative control (Genecefe, Jiangsu, China) in respective cell lines. The sequences of PCDH-RNF32 and RNF32 siRNA were listed in Supplementary Table S1. All transfection steps were carried out as per the instructions of the transfection reagent.

### Quantitative real-time polymerase chain reaction (qRT-PCR)

Total RNA was extracted from the cell lines using Total RNA Extraction Reagent (DP451, Tiangen) following standard protocols. The extracted RNA was used for cDNA synthesis through the cDNA Synthesis Kit (MR05401S, Monad). Gene expression was quantified using SYBR Green Master Mix (MQ10301S, Monad) on a Roche LightCycler 480. The expression levels were calculated by the 2^−ΔΔCT^ method. For normalization, β-Actin and GAPDH were used as an internal reference. The primers used for qRT-PCR amplification were synthesized by Wuhan Jinkairui Bioengineering Co. Ltd. (Wuhan, China). Specifically, the amplification primers for the human RNF32 coding region were TGGGAGAAGGTGAAACAGCG (forward) and TGAAAGCAGCACCTGAGGAC (reverse).

### Western blotting

To perform Western blotting, cells were first lysed with cold RIPA buffer containing PMSF. The isolated total protein was separated by SDS-PAGE and transferred onto PVDF membranes, which were blocked in 5% milk for 2 h and then incubated with primary antibodies against GAPDH (60,004–1-Ig, Proteintech),RNF32 (GTX46903, GeneTex), Bcl-2 (ab182858,Abcam) and Bax (ab32503,Abcam), both diluted to 1:1000, for 1.5 h at 37 °C. Secondary antibodies were then applied and incubated for 1 h at room temperature. Blot development was carried out using ECL Western Blotting Substrate.

### Cell proliferation assays

Cell proliferation was assessed using the Cell Counting Kit-8 (G4103, Servicebio) as per the manufacturer’s guidelines. A total of 0.5 × 10^4^ cells per well were seeded into a 96-well plate for the CCK-8 assay. After 96 h, 10 µl of the Cell Counting Kit solution was added to each well and further incubated at 37 °C for 4 h. The absorbance values at 450 nm were measured with a microplate reader (ELx808, BioTek). The experiment was repeated at least three times to validate the results.

### Migration and invasion assays

For the migration assay, SW480 or Caco2 cells treated with specific agents were added at a concentration of 2.0 × 10^5^ cells/ml to the upper chambers of Transwell inserts (14,341, LABSELECT), while the lower chamber was supplemented with 10% FBS. After 48 h of incubation, the non-migrated cells were discarded, while the migrated cells were stained with 0.1% crystal violet solution. Sections were visualized under an inverted fluorescent microscope (magnification × 200) for downstream analysis.

### Apoptotic cell death assay

Following transfection for 24 h, cells were collected by treating them with EDTA-free trypsin at 300 g and centrifuging them for 5 min at 4 °C. The cells were then washed twice with pre-chilled PBS, each time at 300 g and centrifuged for 5 min at 4 °C. Subsequently, 1–5 × 10^5^ cells were collected and resuspended using 100 μL of L1 × Binding Buffer. Annexin V-647 (5 μL) and PI (5 μL) were added per tube, which was then incubated for 15 min on ice before analyzing immediately using BD Fortessa flow cytometry. The data collected was analyzed using BD FACSDiva™ software (BD Biosciences) and Flowjo v10 software (Flowjo).

### Statistical methods

Wilcoxon test was performed to compare the differences between two groups. Spearman correlation was utilized to calculate correlation coefficients between the risk score and checkpoint genes. Statistical analyses were conducted with R software (4.1.1) and *p* < 0.05 was statistically significant.

## Results

### Immune landscape

**Fig. 1:**
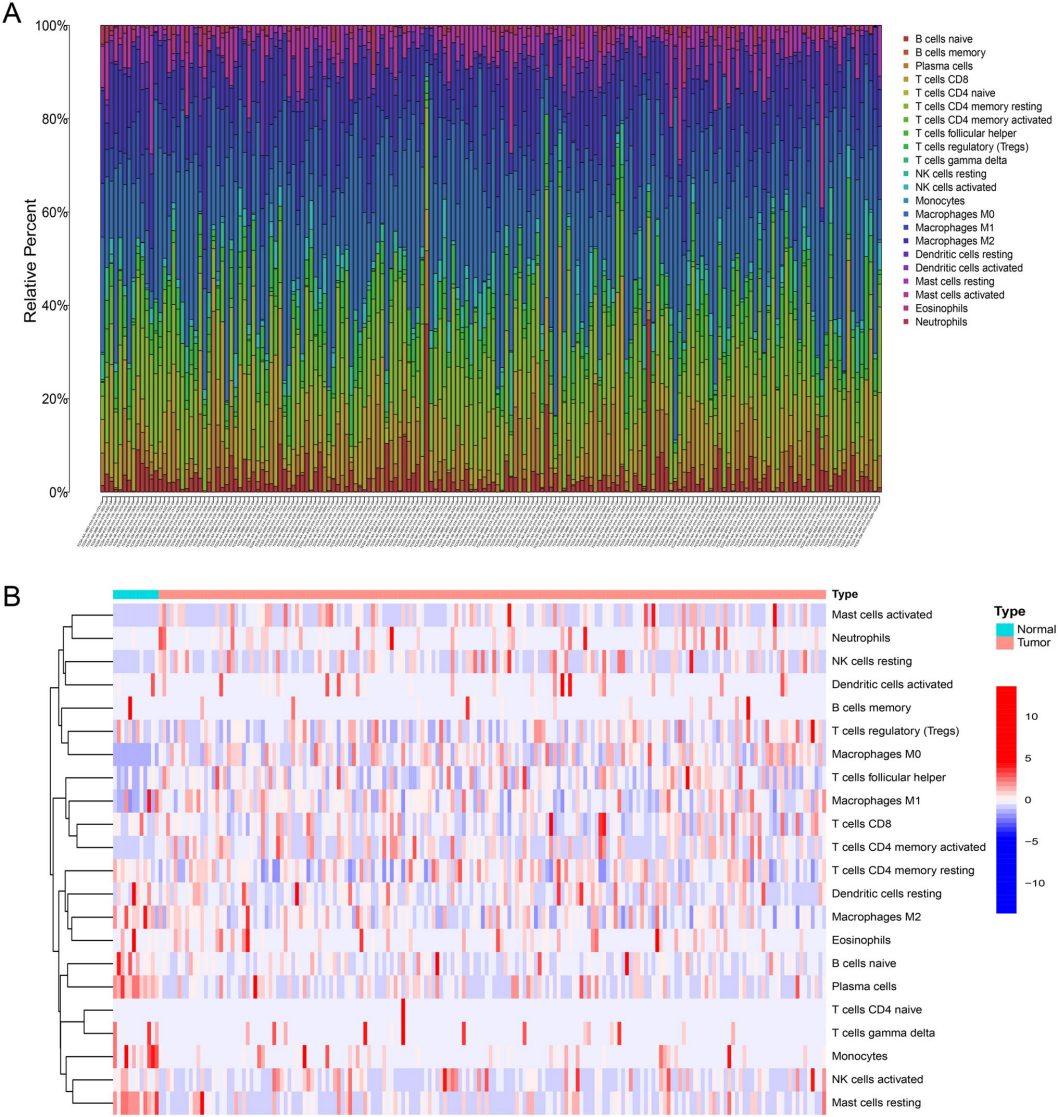
Cluster classification. The Immune landscape of all colon adenocarcinoma (COAD) samples. Different colors indicated different immune cells proportion. **B** Differences of immune cell infiltration between normal and tumor samples

The overall research design is illustrated in Fig. S1. Through CIBERSORT algorithm, immune infiltration scores of every COAD sample were displayed in Table S2. We plotted an immune landscape of all samples and displayed it in Fig. 1A, with different colors indicating different immune cells proportion. Besides, the differences of immune infiltration between normal and tumor samples were displayed in Fig. 1B.

### Screening out M2 macrophage-related genes through WGCNA

**Fig. 2:**
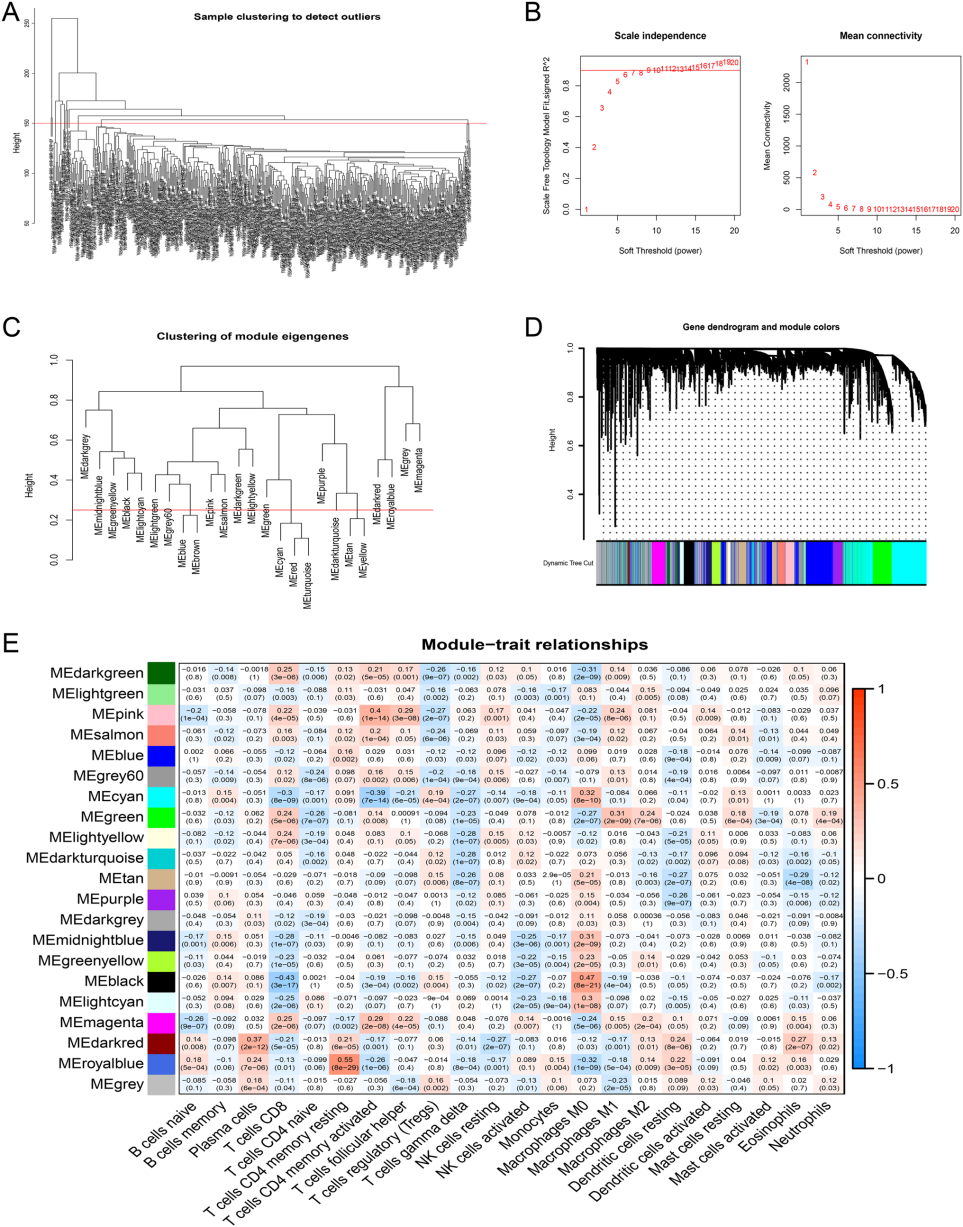
Screening out M2 macrophage-related genes through weighted gene co-expression network analysis (WGCNA). **A** All samples were clustered to detect outliners and 150 was selected as the cutHeight. **B** The optimum soft threshold power was calculated and 6 was selected as the value to build a clustering tree. **C** The module dissection threshold was set as 0.25 to merge similar modules. **D** 21 modules were generated. **E** The correlations between gene modules and immune cells In every module, the above was the correlation coefficient, the below was the *p* value

All samples were clustered to detect outliners and 150 was selected as the cutHeight (Fig. 2A). The optimum soft power was calculated (Fig. 2B) and 6 was selected to merge similar samples and construct a clustering tree (Fig. 2C). The module cutHeight was set as 0.25 to merge similar modules, and thus 21 modules were created (Fig. 2D). The correlations between modules and immune cells were displayed in a heatmap (Fig. 2E). Green and magenta modules containing 1991 genes met the criterion of *p* < 0.001 and were collected for further study.

### Identification of a 7-gene signature

**Fig. 3:**
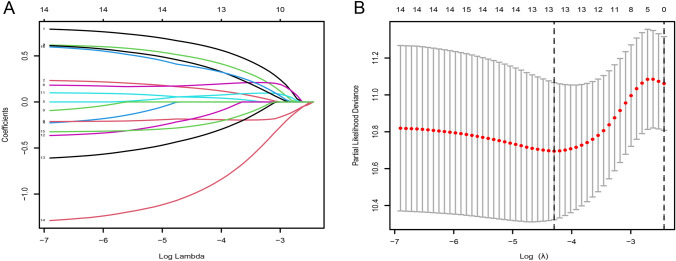
Construction of a prognostic signature. **A**–**B** Through the least absolute shrinkage and selection operator (LASSO) regression, a 7-gene signature was constructed based on the optimum λ

We developed a prognostic signature from 1991 genes identified in the green and magenta modules. Using univariate Cox regression analysis, we have detected 15 genes that are linked to patient overall survival (OS). The results are depicted in Table S3. Subsequently, we constructed prognostic signature for seven genes (FUT11, APOBEC3C, RNF32, NPL, ELOVL3, TNIP3, CD1B) using LASSO (Fig. 3A, B) and multifactorial stepwise Cox regression analyses. The riskscore could be calculated via the gene expression level and coefficients in Table 1.

**Table 1:** Genes involved in the signature and their coefficients

No	Gene	Coefficient
1	FUT11	0.832
2	APOBEC3C	0.263
3	RNF32	0.739
4	NPL	0.625
5	ELOVL3	0.496
6	TNIP3	− 0.691
7	CD1B	− 1.676

### Validation of the risk signature

**Fig. 4:**
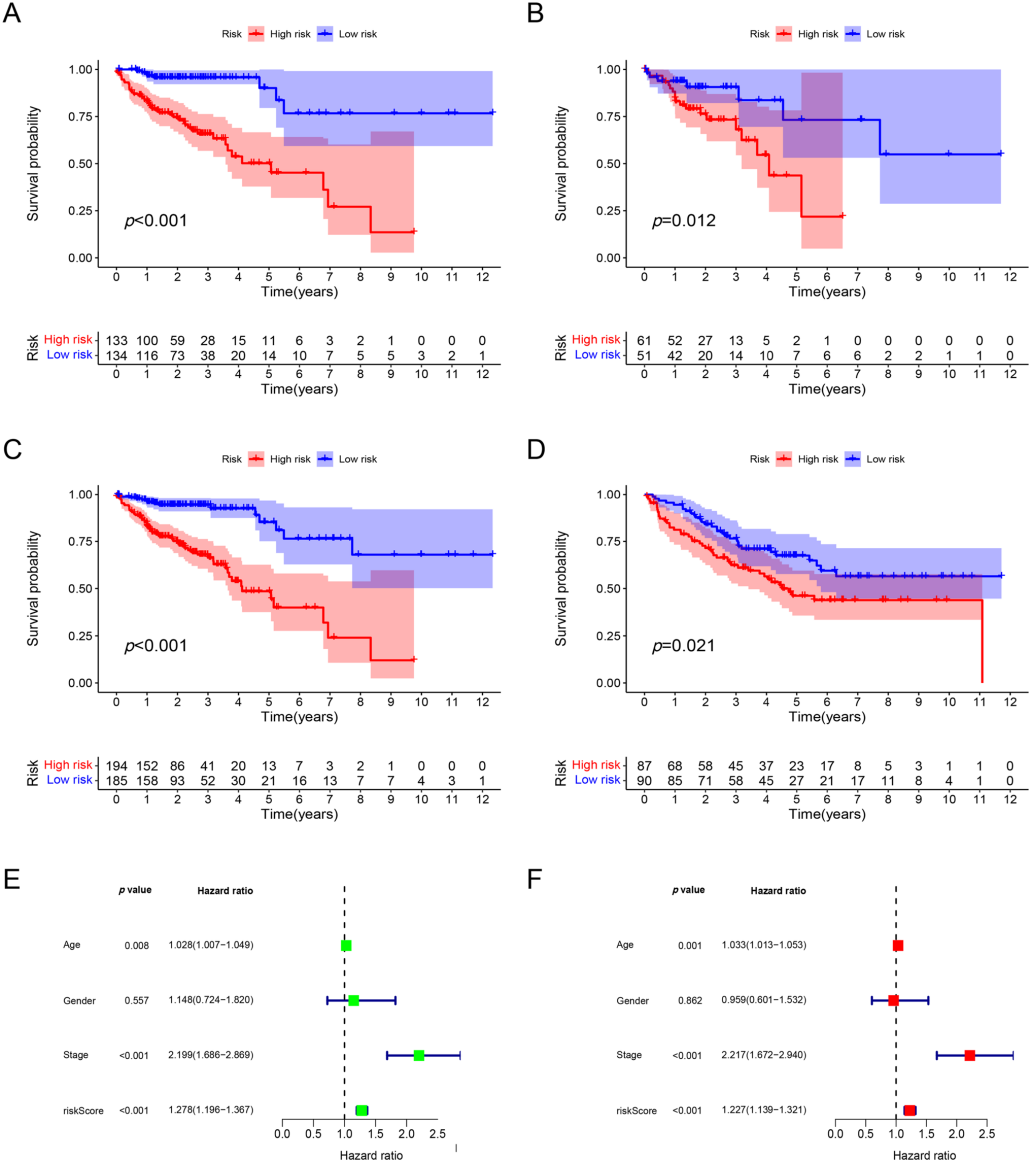
Validation of the risk signature. **A**–**D** Overall survival (OS) of the low-risk group was significantly better than that of the high-risk group in the training TCGA cohort (**A**), the test cohort (**B**), the whole TCGA cohort (**C**) and the GEO cohort (**D**). **E** The univariate regression analysis of the risk score in combination with age, stage, gender in the TCGA cohort. **F** Age, stage, risk score met the criterion of *p* < 0.05 and were incorporated into the multivariate Cox regression analysis

**Fig. 5:**
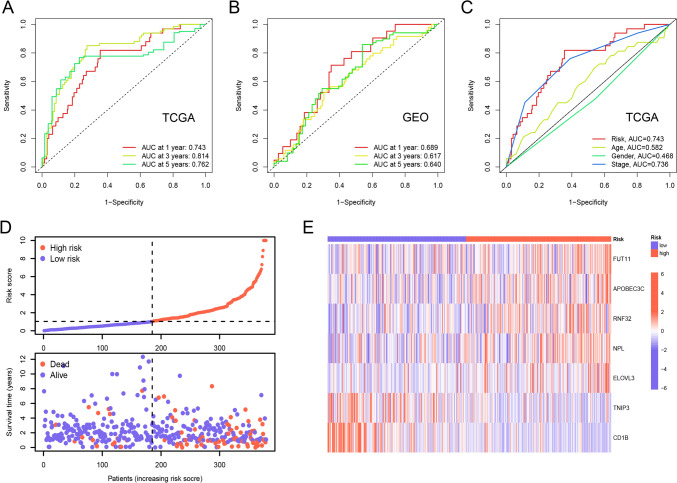
Assess the performance of the risk signature.** A** The area under the ROC curves (AUCs) were separately 0.743 for 1-year, 0.814 for 3-year, and 0.762 for 5-year in the TCGA cohort. **B** The AUCs were separately 0.689 for 1-year, 0.617 for 3-year, and 0.640 for 5-year in the GEO cohort. **C** In the TCGA cohort, the AUC of the signature demonstrates a higher value in comparison to age, gender and stage. **D** As the risk score arose, the number of dead patients increased obviously. **E** The heatmap indicated that the expression levels of five genes in the signature were higher in the high-risk group while those of the other two genes were lower generally

Patients of the training, test, GEO cohorts were separately stratified into low-risk and high-risk groups based on the median riskscore of the training cohort. The KM curves displayed better OS of the low-risk group in the training (Fig. 4A), the test (Fig. 4B), the whole TCGA (Fig. 4C) and the GEO cohorts (Fig. 4D). In the TCGA cohort, both univariate (Fig. 4E) and multivariate (Fig. 4F) Cox proportional hazard analyses revealed age, stage, and risk score as independent prognostic factors. The area under the ROC curves (AUCs) were 0.743, 0.814, 0.762 respectively for 1-, 3-, 5-year in the TCGA cohort (Fig. 5A). The AUCs were separately 0.689, 0.617, 0.640 for 1-, 3-, 5-year in the GEO cohort (Fig. 5B). In the TCGA cohort, the signature exhibits a higher AUC compared to age, gender and stage, as depicted in Fig. 5C.

**Fig. 6:**
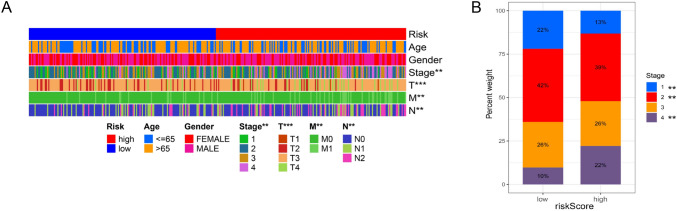
Correlations between riskscore and clinical traits. **A** The differences of stage, T, N and M between high- and low-risk groups were significant. **B** Stage IV accounted for a higher proportion in high-risk group while stage I and II did that in low-risk group. (*p < 0.05, **p < 0.01, ***p < 0.001, ****p < 0.0001)

As the risk score arose, the number of dead patients increased dramatically (Fig. 5D). The heat maps in Fig. 5E illustrate that the expression levels of the genes FUT11, APOBEC3C, RNF32, NPL, and ELOVL3 were notably higher in the high-risk group, whereas the expression levels of the genes TNIP3 and CD1B were predominantly lower. Combined with Fig. 6A, we can find that there are significant differences in tumor stage, T, N, M, between the high-risk group and the low-risk group. In order to more intuitively represent the distribution of tumor stage in the high-risk and low-risk groups, we performed statistics on tumor stage, and found that the difference in stage between the high-risk group and the low-risk group was mainly concentrated in stage I, II, and IV, with a high proportion of stage IV in the high-risk group, and a high proportion of stage I and II in the low-risk group (Fig. 6B).

### Nomogram construction and validation

**Fig. 7:**
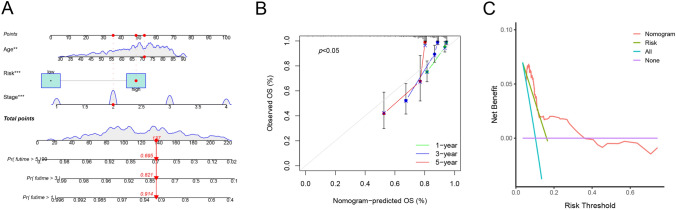
Nomogram construction. **A** A nomogram was constructed to facilitate prognosis prediction. The points of age, stage and risk score were calculated in the nomogram and the total points could indicate the survival probabilities of patients. **B** Calibration curves show that the survival rates predicted by the nomogram differ somewhat from those actually observed. **C** Decision curve analysis of nomogram

With significant clinical characteristics (age and stage), we constructed a nomogram to prognosis prediction (Fig. 7A). The point of age, stage and risk score was calculated with reference to the nomogram and the total points could indicate the survival probabilities of patients. The calibration curves showed that the survival rates predicted by the nomograms differed somewhat from those actually observed (Fig. 7B), which may be related to other confounding factors or insufficient sample size. The results of decision curve analysis (DCA) demonstrated that the nomogram exhibited superior net benefit and a broader threshold probability range for predicting the survival rates of patients (Fig. 7C).

### Immune infiltration differences between groups

**Fig. 8:**
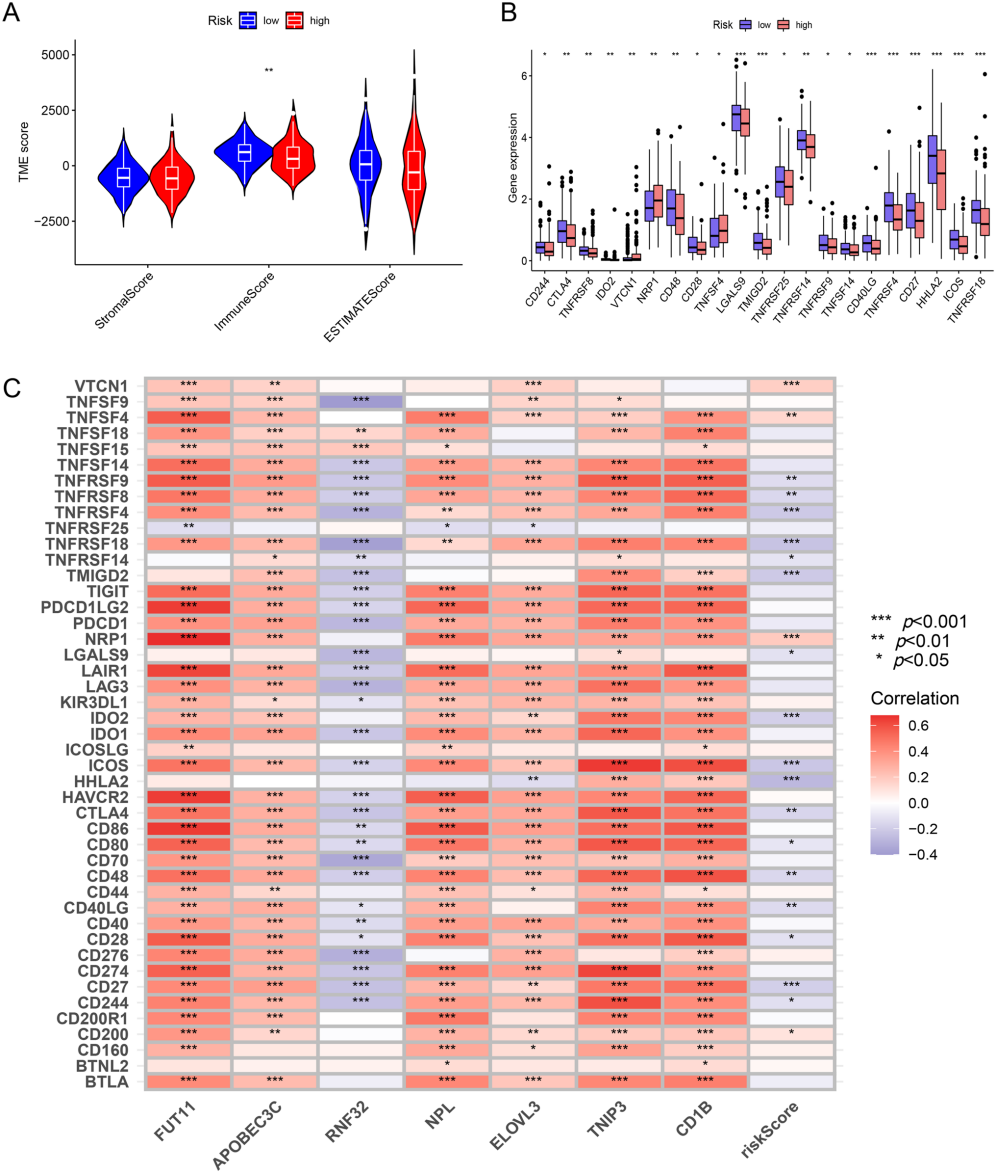
Immune infiltration differences between groups. **A** The immunescore of high-risk group was significantly lower than that of low-risk group. **B** In general, the expression levels of immune-checkpoint genes were higher in low-risk group. **C** The signature was positively correlated with expression levels of four immune-checkpoint genes while negatively correlated with expression levels of 17 immune-checkpoint genes. Six genes in the signature were positively correlated with checkpoint genes except RNF32

The results of immunescore, stromalscore and ESTIMATEscore were listed in Table S4. The immunescore of high-risk group was significantly lower while stromalscore and ESTIMATEscore displayed no significant differences (Fig. 8A). In general, the expression levels of immune-checkpoint genes were lower in high-risk group (Fig. 8B). The riskscore was positively correlated with expression levels of four immune-checkpoint genes while negatively correlated with 17 immune-checkpoint genes. Six genes in the signature displayed positive correlation with checkpoint genes except RNF32 (Fig. 8C).

### Differences of immunotherapy responses between groups

**Fig. 9:**
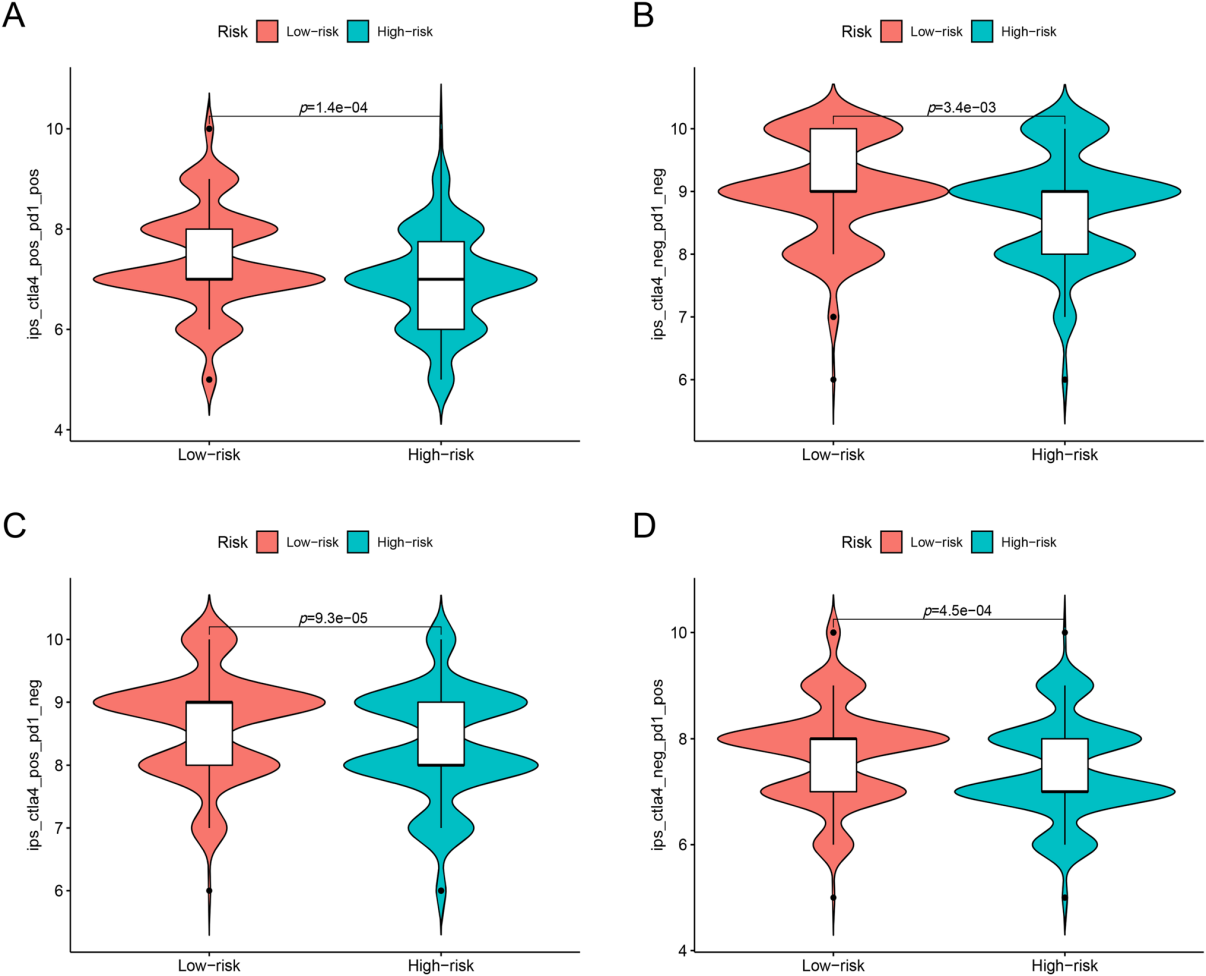
Differences of immunotherapy responses between groups. **A**–**D** The immunophenscores (IPS) of four groups (pos: positive; neg: negative) in low-risk group were significantly higher than those of high-risk group

The IPS of four groups (Table S5) in high-risk group were significantly lower (Fig. 9A-D), indicating lower immunogenicity from immune-checkpoint inhibitors in the high-risk group.

### Validation of risk signature based on GEPIA database

**Fig. 10:**
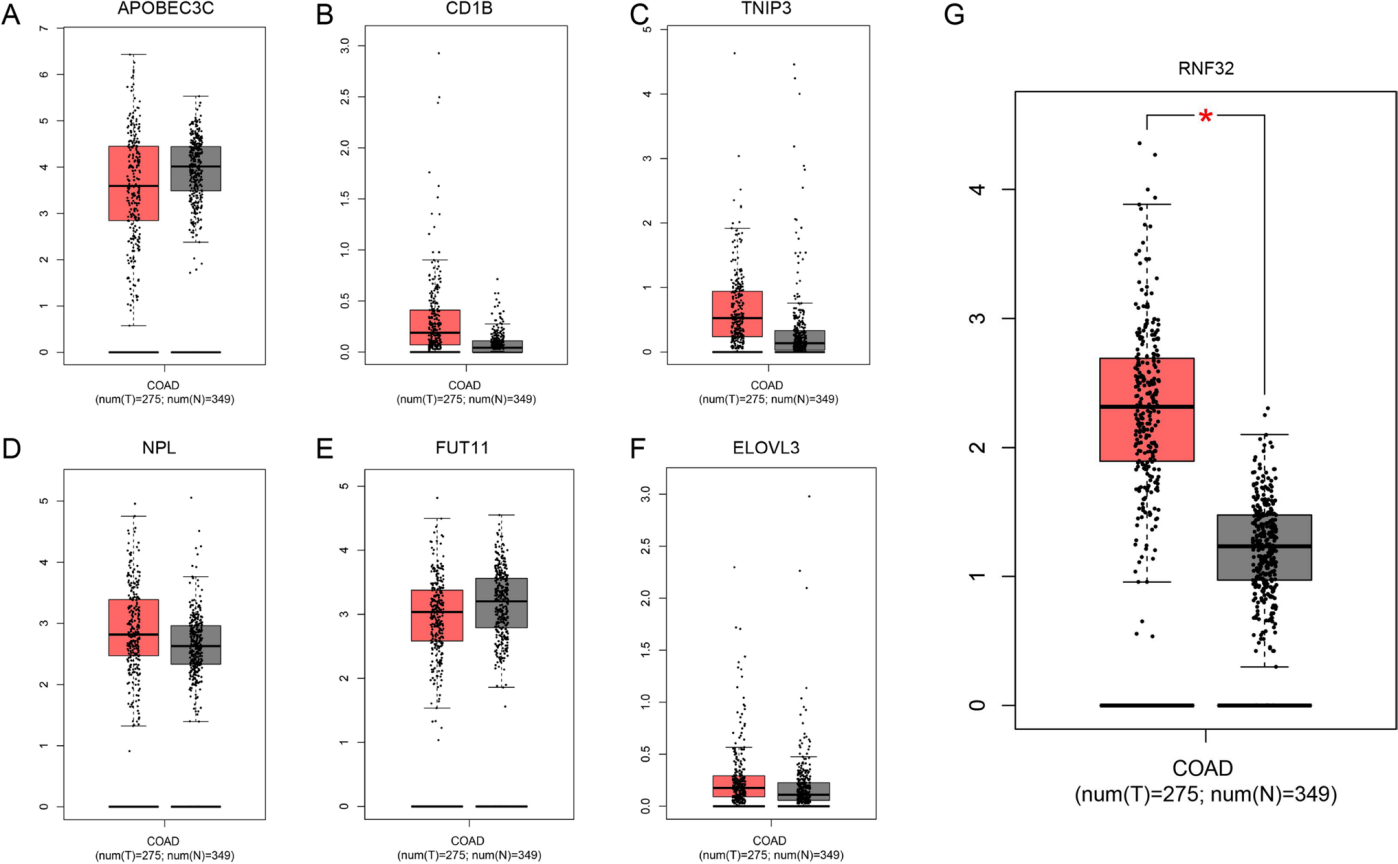
**A**–**G** Using the GEPIA database, expression level of signature genes in COAD compared with normal samples. Red represents tumor samples and gray represents normal samples. (*p < 0.05, **p < 0.01, ***p < 0.001, ****p < 0.0001)

Based on the Gene Expression Profiling Interactive Analysis (GEPIA) databases, we found that there were no significant differences of APOBEC3C, CD1B, TNIP3, NPL, FUT11, ELOVL3 in COAD samples compared to normal samples. However, the expression of RNF32 was significantly higher in COAD samples (Fig. 10A-G).

### Validation of RNF32 expression and biological function in colon cancer cells

**Fig. 11:**
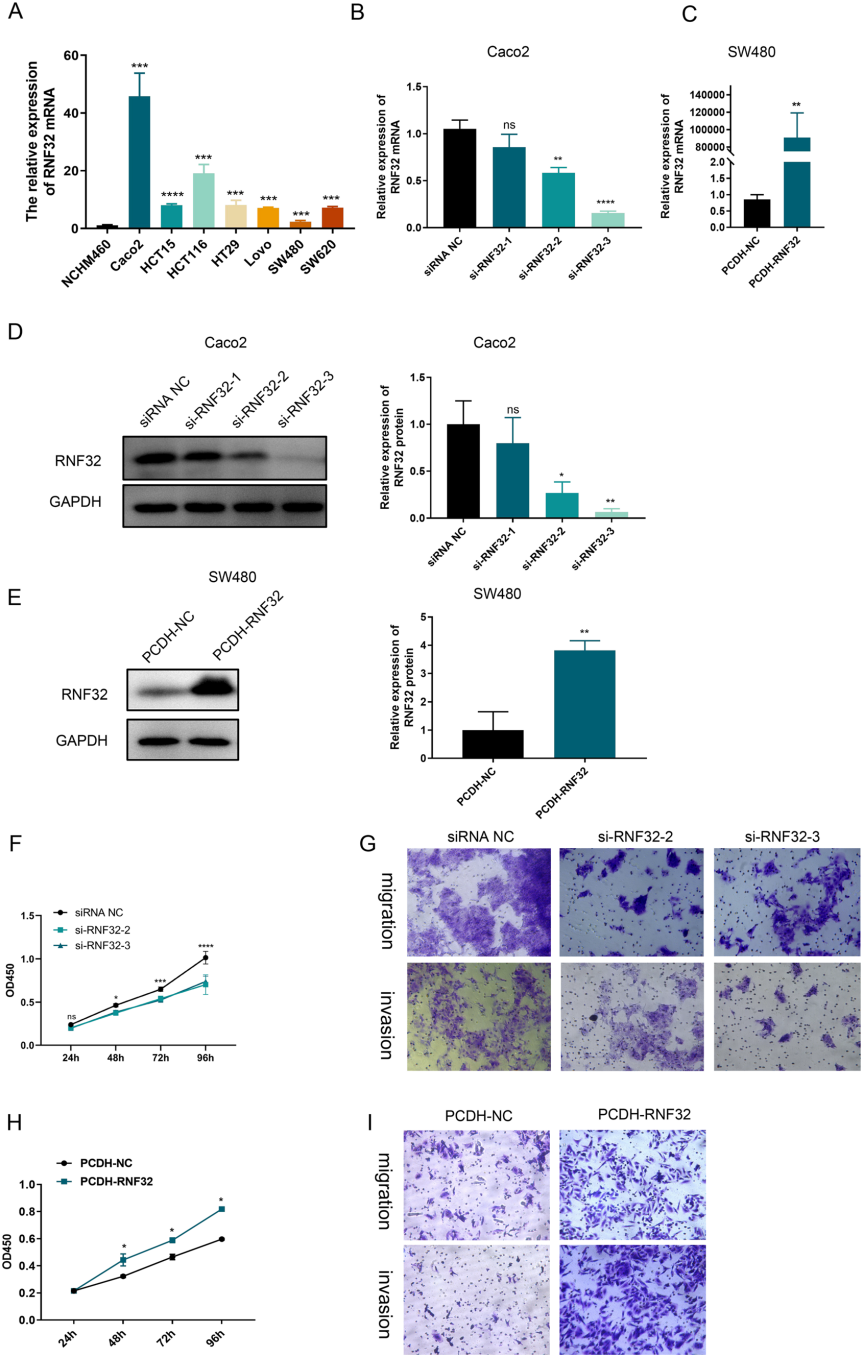
**A** qRT-PCR analysis of RNF32 expression differences in colon cancer cell lines. **B**–**C** qRT-PCR analysis of RNF32 expression in colon cancer cells after knockdown and overexpression of the gene. **D**–**E** Western Blot analysis of RNF32 expression in colon cancer cells after knockdown and overexpression of the gene. **F, H** Effect of knockdown and overexpression of RNF32 on proliferative capacity of colon cancer cells. **G, I** Effect of knockdown and overexpression of RNF32 on the migration and invasion ability of colon cancer cells. (*p < 0.05, **p < 0.01, ***p < 0.001, ****p < 0.0001)

Based on above findings, RNF32 had a significantly higher expression level in COAD samples, implying a probable involvement in the development or progression of COAD that necessitates further validation. Therefore, we further examined the expression and function of RNF32 in vitro research. As shown in Fig. 11A, the expression profile of RNF32 was significantly elevated in different colon cancer cell lines when compared to that of normal intestinal epithelial NCHM460 cells. Moreover, we investigated the effects of knockdown and overexpression of RNF32 expression in colon cancer cell lines (Caco2 and SW480). The knockdown efficiency of three si-RNA32 sequences was assessed in Caco2 cells using qRT-PCR (Fig. 11B). Our data revealed that si-RNA32-2 and si-RNA32-3 exhibited the most substantial inhibitory effect on RNF32 expression. After transfecting SW480 cells with the PCDH-RNA32 overexpression plasmid, we observed a significant increase in the mRNA levels of RNA32, as demonstrated in Fig. 11C. The Western blot analysis revealed that the knockdown of RNF32 prominently reduced the expression level of RNF32 protein in colon cancer cells, whereas the overexpression of RNF32 resulted in a significant increase in RNF32 protein expression in those cells (Fig. 11D–E).

**Fig. 12:**
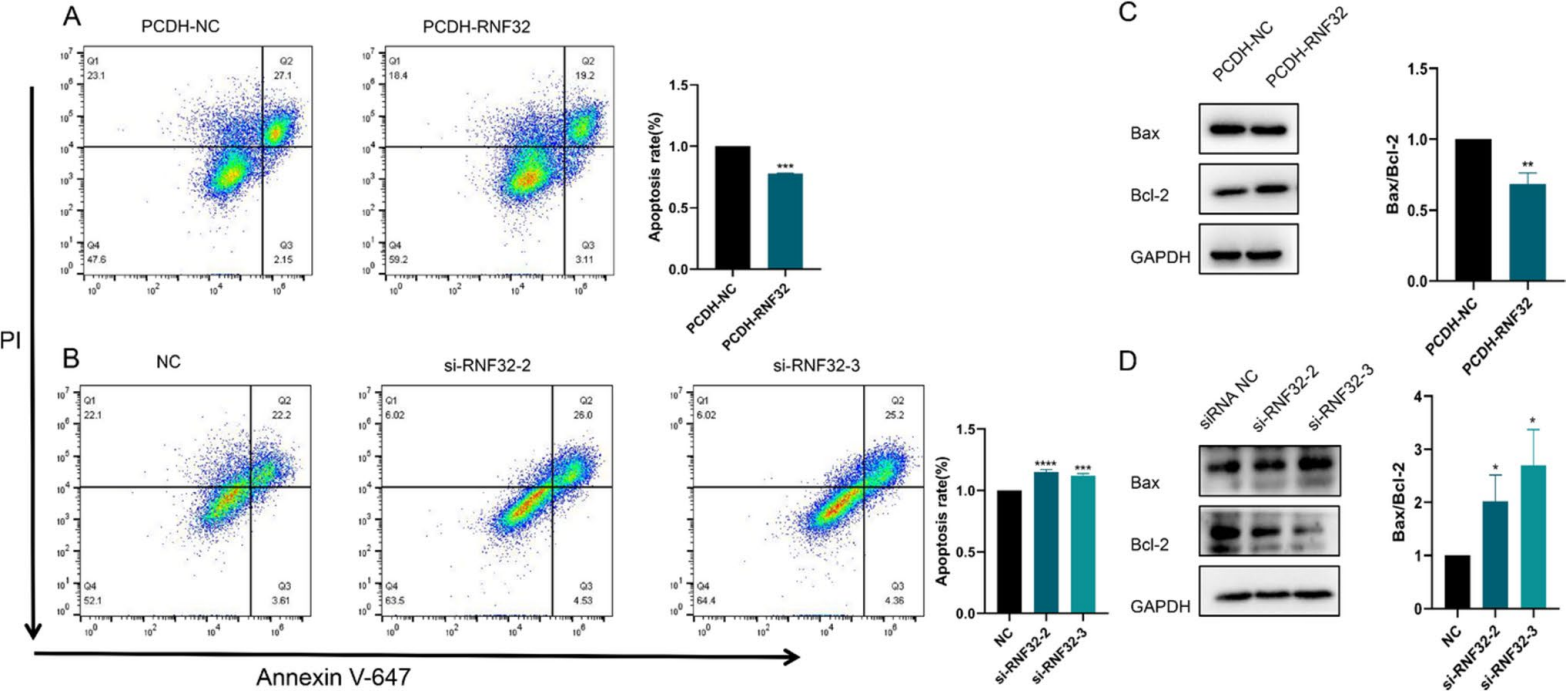
**A** Impact of overexpression of RNF32 on the level of apoptosis in colon cancer cells. **B** Effect of knockdown of RNF32 on the level of apoptosis in colon cancer cells. **C** Effect of overexpression of RNF32 on Bax/Bcl-2 apoptotic protein in colon cancer cells. **D** Effect of knockdown of RNF32 on Bax/Bcl-2 apoptotic protein in colon cancer cells. (*p < 0.05, **p < 0.01, ***: *p* < 0.001, ****p < 0.0001)

To expand our comprehension of RNF32's biological role in COAD, we conducted experiments exploring the impacts of RNF32 knockdown and overexpression on Caco2 and SW480 cells' biological behavior. The cell viability of Caco2 cells was substantially reduced upon transfection with si-RNF32-2,3 relative to si-NC-transfected cells (Fig. 11F). Similarly, migration, proliferation, and invasion capacity drastically declined in these cells (Fig. 11G). On the contrary, SW480 cells transfected with PCDH-RNF32 exhibited pronounced cell viability improvement compared to PCDH-NC-transfected cells (Fig. 11H), and there was significant enhancement of cell migration, proliferation, and invasion capacity as well (Fig. 11I). In addition, our study also found that overexpression of RNF32 inhibited apoptosis and the level of Bax/Bcl-2 apoptotic protein expression was significantly decreased in cancer cells (Fig. 12A, C), whereas knockdown of RNF32 enhanced apoptosis and the level of Bax/Bcl-2 apoptotic protein expression was significantly increased in cancer cells (Fig. 12B, D). Thus, RNF32 may promote the proliferation, migration and invasion of colon cancer cells by inhibiting apoptosis.

## Discussion

As the cardinal roles in tumor immunosuppression, M2 macrophage were revealed structurally and functionally activated in various cancers. Extensive evidence has underlined the importance of M2 macrophage in tumor progression in various cancers. For instance, M2 macrophage-secreted CHI3L1 activated IL-13Rα2 expression of gastric cancer cells and advanced the metastasis (Chen et al. 2017). In Colon Cancer, M2 macrophage induced colorectal tumor cells migration through macrophage–generated exosomes which downregulate BRG1 expression (Lan et al. 2019). In vivo, the tumor secreted secretory protein cathepsin K which could stimulate the M2 polarization and thereby facilitate the progression of CRC (Li et al. 2019). Also, M2 macrophage was also found to related to the therapeutic responses to ICIs which have achieved great success in cancer immunotherapies. For instance, repolarization M2 tumor-associated macrophages to M1 macrophages was also found to potentiate the anticancer efficacy of ICIs (Choo et al. 2018).

So far, the roles of M2 macrophage in carcinogenesis and immunotherapies have not been elucidated completely because of the complexity and the heterogeneity in different contexts. Thus, integrative analysis of M2 macrophage at a transcriptome level could advance the research. In this study, we aimed to screen out M2 macrophage-related genes and investigate the clinical implication of these genes in COAD through transcriptome sequencing data. Notably, the riskscore based on M2 macrophage-related genes were negatively related to immune infiltration and immune-checkpoint genes. In particular, expression levels of immune-checkpoint genes were generally higher in low-risk group, which indicates the protective role of the immune infiltration and was consistent with previous studies (Disis 2010; Waldner et al. 2006). Also, the signature could stratify patients who were more inclined to response to ICIs. And thereby the signature might be an indicator of immune infiltration and responses in COAD.

Given that M2 macrophage was correlated with the poor outcome of patients in multiple solid tumors such as colorectal cancer (Edin et al. 2012), non-small-cell lung cancer (Cao et al. 2019), pancreatic cancer (Hu et al. 2016). To assess the potential of M2 macrophage-related genes as panels of biomarkers for OS prediction of COAD patients, we also constructed a signature comprising of seven genes. To evaluate the precision of the signature, we conducted both internal and external validation. The efficacy of the signature was excellent in two cohorts, and besides the risk stratification showed much consistency with clinical stage. Together, all this provided a novel tool for prognosis prediction and added new evidence of the mysterious roles of M2 macrophage in the context of COAD microenvironment at a transcriptome level.

In the signature, most genes positively contributed to the riskscore, which indicated their roles as oncogenes. Fucosyltransferase 11 (FUT11), activated by transcription factor HIF1α, advanced the proliferation and mobility of hepatocellular carcinoma cells (Ruan et al. 2021). In a meta-analysis, expression of FUT11 in renal cell carcinoma was positively related to disease progression (Zodro et al. 2014). The aberrant upregulation of members in ring finger protein family, as cancer/testis genes, was associated with colon cancer progression and migration (Wang et al. 2016; Wei et al. 2020). Our results also suggested the same role of ring finger protein 32 (RNF32) in COAD. CD1B was cell surface glycoprotein associated with antigen presentation function and involves in both innate and adaptive immune responses. Previous study in localized prostate cancer also indicated that low CD1B expression could indicate poorer recurrence-free survival (Lee et al. 2019). Currently, there are few researches available about the roles of these genes in COAD and our research might provide some evidences.

Our subsequent experimental investigations revealed that RNF32 is significantly overexpressed in colon cancer cells, RNF32 may promote the proliferation, migration, and invasion of colon cancer cells by inhibiting apoptosis. This observation aligns with previous studies linking the RNF32 to the pathogenesis of esophageal cancer from Barrett’s esophagus (Wang et al. 2014). However, given the scarcity of research regarding RNF32’s involvement in colon cancer, our findings offer valuable insight into the potential of RNF32 as a novel therapeutic target for COAD patients.

Overall, our study contributed to the identification of M2 macrophage-related genes and proposed a novel transcriptome-based approach to predicting COAD prognosis. Moreover, we assessed the biomarker’s potential in immunotherapy. Nonetheless, our research has limitations and shortcomings, including a lack of comprehensive understanding of RNF32’s functional phenotype, and future experiments involving animal models are necessary to address these issues.

## Conclusion

In this study, the division into different risk groups based on M2 macrophage-related genes could stratify patients accurately. Also, the risk score was negatively correlated with immune infiltration. Moreover, the signature could provide some references for precise immunotherapy.

### Supplementary Information

Below is the link to the electronic supplementary material.

Supplementary file1 (DOCX 9306 KB)

Supplementary file2 (DOCX 11 KB)

Supplementary file3 (XLSX 133 KB)

Supplementary file4 (DOCX 13 KB)

Supplementary file5 (XLSX 39 KB)

Supplementary file6 (XLSX 21 KB)

## Data Availability

The data are available from TCGA database (https://portal.gdc.cancer.gov/), the GEO database (http://www.ncbi.nlm.nih.gov/geo/, GSE17536) and TCIA website (https://tcia.at/home).

## Abbreviations

COAD: Colon adenocarcinoma
TCGA: The cancer genome atlas
GEO: Gene expression omnibus
WGCNA: Weighted gene co-expression network analysis
GEPIA: Gene expression profiling interactive analysis
LASSO: Least absolute shrinkage and selection operator
PI: Propidiumlodide
IPS: Immunophenscores
TME: Tumor microenvironment
TNM: Tumor node metastasis
T: Tumor
N: Node
M: Metastasis
OS: Overall survival
AUC: Area under curve
ROS: Receiver operating characteristic
Bax: Bcl-2-associated X
Bcl-2: B-cell lymphoma-2
