# Supplementary figures and images for: Prognosis and immunotherapy response prediction based on M2 macrophage-related genes in colon cancer
Source: J Cancer Res Clin Oncol. 2024 Jan 25;150(2):31. doi: 10.1007/s00432-023-05573-6 (PMC10811099; doi:10.1007/s00432-023-05573-6)

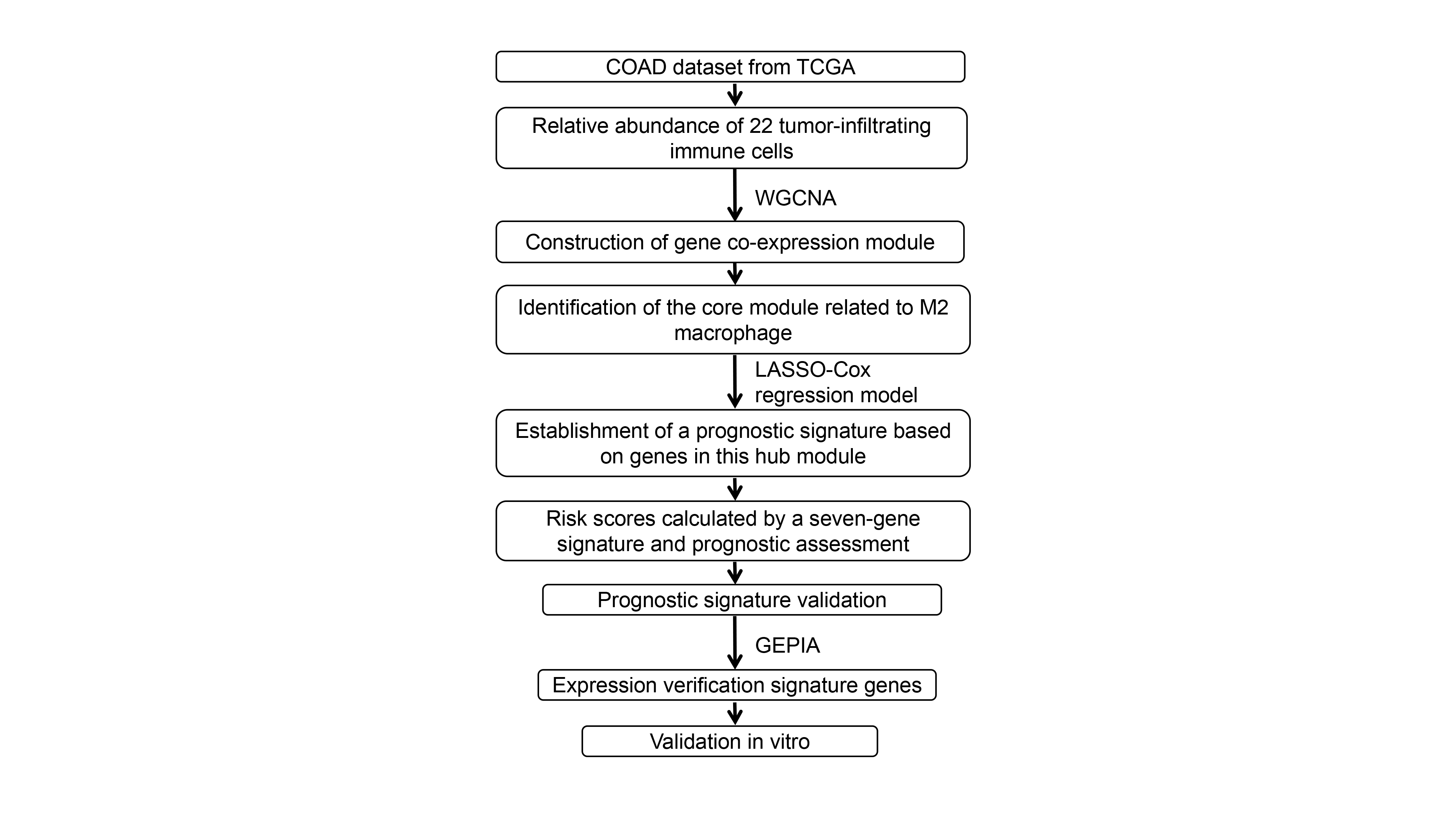


**Figure S1.** Data collection and analysis in this study.

Supplement: Supplementary file 1 — Supplementary file1 (DOCX 9306 KB) [file 432_2023_5573_MOESM1_ESM.docx]
