# Supplementary material for: Prognosis and immunotherapy response prediction based on M2 macrophage-related genes in colon cancer
Source: J Cancer Res Clin Oncol. 2024 Jan 25;150(2):31. doi: 10.1007/s00432-023-05573-6 (PMC10811099; doi:10.1007/s00432-023-05573-6)
Supplement: Supplementary file 2 — Supplementary file2 (DOCX 11 KB) [file 432_2023_5573_MOESM2_ESM.docx]

Table S1: Sequence of PCDH-RNF32 and RNF32 siRNAs.

| Name | Sequences |
| --- | --- |
| si-NC | ACGTGACACGTTCGGAGAA |
| si-RNF32-1 | CATCAGATGAATGGGAGAAGGTGAA |
| si-RNF32-2 | CCAAGCCTACTGGAGAGGATGTGTT |
| si-RNF32-3 | CCAGAAGAAGATTCTTGAATGTTGA |
| PCDH-RNF32 | ATGTTAAAAAATAAGGGTCACTCATCTAAGAAAGATAACTTGGCAGTCAATGCAGTTGCTTTACAAGATCACATTTTACATGATCTTCAACTTCGAAATCTTTCAGTTGCAGATCATTCTAAGACACAAGTACAAAAGAAAGAGAACAAATCTCTAAAAAGAGATACAAAGGCAATAATAGATACTGGACTTAAAAAAACTACACAGTGCCCCAAACTAGAAGACTCAGAAAAAGAATATGTTCTTGATCCCAAACCGCCGCCGTTGACTTTGGCACAGAAGTTGGGCCTCATTGGGCCTCCACCACCTCCACTGTCATCAGATGAATGGGAGAAGGTGAAACAGCGCTCTCTCCTGCAAGGGGACTCCGTGCAACCATGCCCCATCTGTAAAGAAGAATTCGAGCTTCGTCCTCAGGTGCTGCTTTCATGCTCCCATGTGTTCCACAAAGCATGTCTTCAGGCTTTTGAAAAGTTCACAAATAAGAAAACCTGTCCTCTCTGTAGAAAGAACCAGTATCAAACCCGAGTGATACACGATGGGGCCCGCCTGTTCAGAATCAAGTGTGTGACCAGAATCCAAGCCTACTGGAGAGGATGTGTTGTTAGAAAGTGGTACAGAAACCTGAGGAAAACAGTACCTCCCACAGATGCCAAGTTAAGAAAAAAATTCTTTGAAAAAAAGTTCACAGAAATCAGCCACCGCATCCTGTGCTCATACAACACCAACATTGAAGAGCTCTTTGCAGAAATCGATCAGTGCTTGGCCATAAATCGAAGTGTTCTTCAGCAGTTGGAAGAAAAATGTGGCCATGAGATCACAGAAGAGGAATGGGAGAAAATCCAAGTGCAGGCTCTGCGCCGGGAGACCCACGAGTGCTCCATCTGCCTGGCCCCTCTCTCCGCTGCTGGCGGTCAGCGCGTGGGTGCAGGCAGGCGTTCCAGAGAGATGGCCCTCCTGTCCTGCTCACATGTGTTCCACCATGCGTGTCTGCTGGCACTAGAGGAGTTCTCCGTGGGAGACAGGCCTCCTTTCCATGCCTGTCCTCTCTGCCGCTCCTGCTACCAGAAGAAGATTCTTGAATGTTGA |
