# Supplementary material for: Prognosis and immunotherapy response prediction based on M2 macrophage-related genes in colon cancer
Source: J Cancer Res Clin Oncol. 2024 Jan 25;150(2):31. doi: 10.1007/s00432-023-05573-6 (PMC10811099; doi:10.1007/s00432-023-05573-6)
Supplement: Supplementary file 4 — Supplementary file4 (DOCX 13 KB) [file 432_2023_5573_MOESM4_ESM.docx]

**Table S3. Univariate Cox analysis of the TCGA cohort.**

| **GENE ID** | **HR** | **HR.95L** | **HR.95H** | **P value** |
| --- | --- | --- | --- | --- |
| FUT11 | 1.929727 | 1.099571 | 3.386633 | 0.021979 |
| APOBEC3C | 1.332528 | 1.015784 | 1.748038 | 0.038167 |
| RNF32 | 1.922949 | 1.160291 | 3.186902 | 0.011189 |
| GJA3 | 1.598663 | 1.037482 | 2.46339 | 0.033439 |
| CSTL1 | 1.904821 | 1.219936 | 2.974207 | 0.004591 |
| APOBEC3F | 1.781999 | 1.11502 | 2.84795 | 0.015732 |
| NPL | 1.571312 | 1.007992 | 2.449444 | 0.046031 |
| CCL22 | 0.62975 | 0.462123 | 0.858182 | 0.003406 |
| DENND6B | 1.802894 | 1.000415 | 3.249079 | 0.049839 |
| ELOVL3 | 1.551336 | 1.030677 | 2.335012 | 0.035309 |
| ULBP1 | 1.840592 | 1.165871 | 2.905793 | 0.008826 |
| TEX11 | 0.252042 | 0.068423 | 0.928415 | 0.038301 |
| TNIP3 | 0.585631 | 0.347086 | 0.988124 | 0.044992 |
| CD1B | 0.340374 | 0.165542 | 0.699851 | 0.003386 |
| TRAP1 | 0.506866 | 0.282909 | 0.908112 | 0.022375 |
